# *In Vivo* Evaluation of Immediately Loaded Stainless Steel and Titanium Orthodontic Screws in a Growing Bone

**DOI:** 10.1371/journal.pone.0076223

**Published:** 2013-10-04

**Authors:** Kerstin Gritsch, Norbert Laroche, Jeanne-Marie Bonnet, Patrick Exbrayat, Laurent Morgon, Muriel Rabilloud, Brigitte Grosgogeat

**Affiliations:** 1 Laboratoire des Multimatériaux et Interfaces (UMR CNRS 5615), Faculté d′Odontologie de Lyon, Université de Lyon, Lyon, France; 2 Hospices Civils de Lyon, Lyon, France; 3 Laboratoire de Biologie Intégrative du Tissu Osseux (INSERM U890), Faculté de Médecine, Université Jean Monnet, Saint-Etienne, France; 4 Agressions Vasculaires et réponses tissulaires (INSERM ERI22/EA4173), Université de Lyon, Lyon, France; 5 Laboratoire Biostatistique Santé (UMR CNRS 5558), Université de Lyon, Lyon, France; Université de Technologie de Compiègne, France

## Abstract

The present work intends to evaluate the use of immediate loaded orthodontic screws in a growing model, and to study the specific bone response. Thirty-two screws (half of stainless steel and half of titanium) were inserted in the alveolar bone of 8 growing pigs. The devices were immediately loaded with a 100 g orthodontic force. Two loading periods were assessed: 4 and 12 weeks. Both systems of screws were clinically assessed. Histological observations and histomorphometric analysis evaluated the percent of “bone-to-implant contact” and static and dynamic bone parameters in the vicinity of the devices (test zone) and in a bone area located 1.5 cm posterior to the devices (control zone). Both systems exhibit similar responses for the survival rate; 87.5% and 81.3% for stainless steel and titanium respectively (p = 0.64; 4-week period), and 62.5% and 50.0% for stainless steel and titanium respectively (p = 0.09; 12-week period). No significant differences between the devices were found regarding the percent of “bone-to-implant contact” (p = 0.1) or the static and dynamic bone parameters. However, the 5% threshold of “bone-to-implant contact” was obtained after 4 weeks with the stainless steel devices, leading to increased survival rate values. Bone in the vicinity of the miniscrew implants showed evidence of a significant increase in bone trabecular thickness when compared to bone in the control zone (p = 0.05). In our study, it is likely that increased trabecular thickness is a way for low density bone to respond to the stress induced by loading.

## Introduction

Endosseous metallic screws are widely used in orthopedics, maxillofacial surgery and more recently in orthodontics, as these devices provide a good clinical control of tooth movement in adolescents. Screws are temporarily fixed to the alveolar bone and removed after treatment 1. Furthermore, they can be immediately loaded without a healing period 2–6. Thus, if mechanical stability is needed to withstand the applied orthodontic forces, osseointegration needs to be avoided. Nonetheless, screws stability is related to the amount of bone contact 7. Most orthodontic screws are made of titanium (or titanium alloys), known to allow for osseointegration. Even in cases of immediate loading, titanium-made orthodontic screws can achieve partial osseointegration after 3 weeks 8,9., or randomly organized osseointegration islets after longer periods 10. For orthopedic devices, decreased bone interface is found around stainless steel screws compared to titanium screws 11. The aim of this study is to evaluate the use of stainless steel screws in place of titanium in orthodontic indications, and to study the specific growing bone response in the vicinity of the devices. The objective of the experiment is to evaluate the survival rate, the mechanical stability and the tissular response around both systems of miniscrew implants (titanium alloy and stainless steel), following immediate loading in growing pigs.

## Materials and Methods

### Ethics statement

The protocol of this animal study was approved by the ethical committee on animal research at the National Veterinary School of Lyon in France. The ARRIVE guidelines were used as a reference to prepare this manuscript.

### Devices

**Figure 1:**
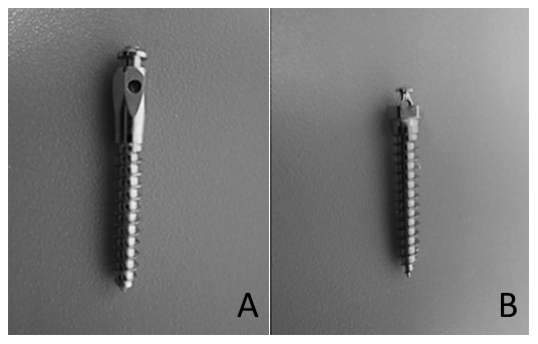
Screws evaluated in the present study; A. Leone (stainless steel, 2 mm in diameter) B. Absoanchor (Ti6Al4V, 2-1.8 mm in diameter).

The screws studied are stainless steel (Leone; Firenze, Italy) and titanium-aluminium-vanadium orthodontic screws (Ti6Al4V, Absoanchor®; Dentos, Daegu, Korea); both are 2 mm in diameter and 10 or 12 mm in length (Fig.1). The stainless steel devices are cylindrical and the Ti6Al4V devices are conic-shaped.

### Surgical procedure

**Figure 2:**
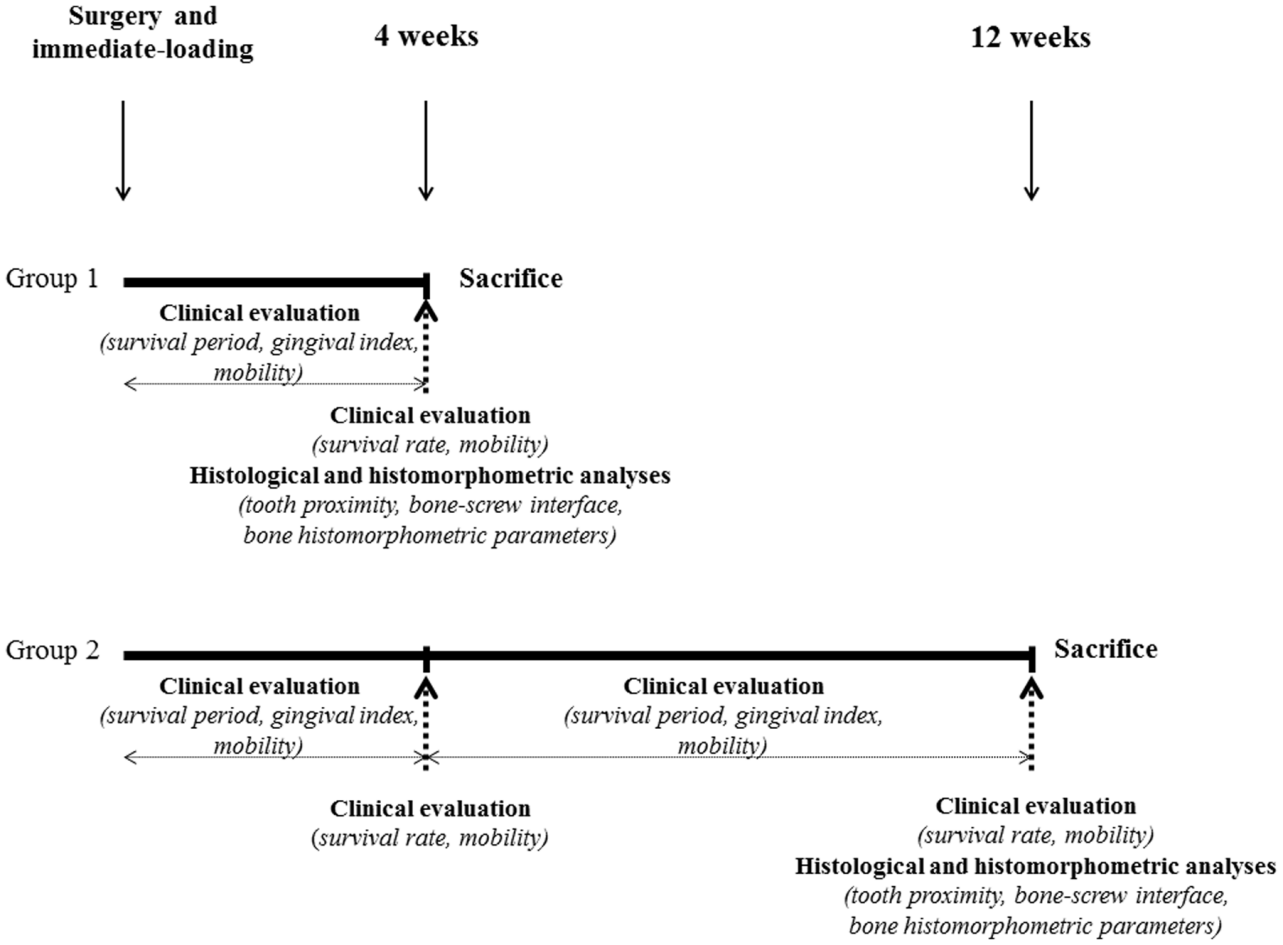
Study design.

Eight three-month-old Large White pigs (26–30 kg) were considered. Prior to the surgery, the animals were conditioned in boxes (2 per box), where they remained during a one-week observation period. The surgical procedure was conducted under general anesthesia performed by an intravenous injection of propofol (Diprivan®, 4–5 mg/kg for induction and 20 mL/h for maintenance). After a flap was raised, a pilot hole was drilled through the cortical bone (1.7 mm diameter drill) using a contra-angle low-speed implant handpiece. On each animal, 4 screws (2 per side) were inserted in the mandible: one in an anterior location (12 mm length, 2–3 mm posterior to the canine) and the other in a posterior location (10 mm length, between the first and the second molars). For both locations, the devices were inserted on the buccal side (2–3 mm from the teeth cervix), in the attached gingiva, with 10° to 20° angulation to the alveolar process. The same screws (stainless steel or titanium) were inserted in the same quadrant (randomly assigned). After checking the primary stability with dental tweezers, the devices were immediately loaded with nickel-titanium coil springs through a bonded bracket on adjacent teeth and a 100 g load was applied. Antibiotics (amoxicillin Clamoxyl®– intramuscular injection, 10 mg/kg/day) and analgesics (ketoprofen Ketofen®- intramuscular injection, 3 mg/kg) were given for 7 postoperative days. The animals were monitored daily for pain and infection during this postoperative period. Two loading periods were assessed: 4 weeks and 12 weeks (Fig.2). The pigs were randomly divided into two groups: four animals (group 1) were killed 4 weeks after the surgery; the four others (group 2) were sacrificed 12 weeks after the surgery. For the 4-week loading period, clinical evaluation was performed for the eight animals (group 1 and group 2) as this assessment did not require the animals to be sacrificed; histological analysis was performed on the four animals of group 1 only, as this last assessment required bone samples. For the 12-week loading period, clinical evaluation and histological analysis were performed on the four animals of group 2. The animals were fed *ad libidum* with a soft diet, and an oral hygiene program was put in place: following sedation (Zoletil®, Virbac, Carros, France - intramuscular injection, 6 mg/kg), miniscrew implants and peri-implant areas were manually brushed twice a week with individual toothbrushes (Inava 7/100e, Pierre Fabre, Castres, France) and oxygenated water (10 vol.). A daily veterinary follow-up was performed to control the animals' general health and welfare. Bone labeling was performed by intramuscular injections of tetracycline (20 mg/kg) 10 and 9 days, and 2 and 1 days before death. Sacrifice was performed by lethal intravenous injection of T61® (Hoechst Roussel Vet, Pantin, France – intravenous injection, 4-6 mL/50 kg) after tranquillization with Zoletil®.

### Clinical analysis

The survival rate of the devices, dental plaque, gingival inflammation and mobility (dental tweezers) were noted twice a week. Gingival inflammation was assessed by means of a gingival index, 0) no gingival inflammation, 1) slight inflammation, 2) moderate inflammation, 3) severe inflammation. The mean gingival inflammation index was calculated from the scores obtained for each oral hygiene session during the entire loading period.

### Histological preparation and histomorphometric analysis

**Figure 3:**
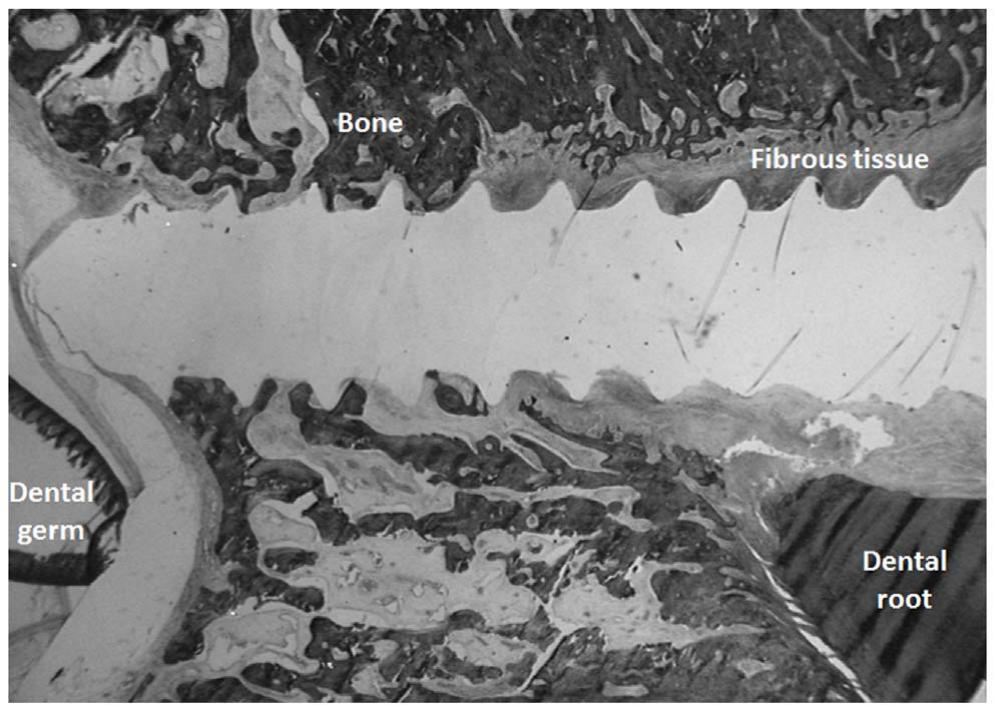
Histological slide (Modified Goldner's Masson Trichrome) of a screw device (Group 1: stainless steel device, posterior area) (x1.6); proximity with germ and root can be noticed.

Immediately following removal, the mandibles were fixed in a 10% formaldehyde solution for 48 hours and then maintained in acetone at 4°C until further processing. Bone samples (containing the devices and their surrounding tissues) were removed from the sectioned pig mandibles. Screws were pulled out with the appropriate screwdriver before the histological analysis. The undecalcified bone samples were embedded in methylmetacrylate at 4°C according to a previously described protocol 12. and 9 µm-thick slices were prepared with a microtome (PolycutS, Leica, Wetzlar, Germany). Parallel sections to the long axis of the screws were performed so that buccal and lingual bone areas were to the left and the right of the screws on the histological sections. The slices were stained with a Modified Goldner's Masson Trichrome solution and observed under an optical microscope (DMRB microscope, Leica, Wetzlar, Germany). Since contact between the devices and dental germs or roots is considered to be a cause of failure, in this orthodontic use, the relationship between the screws and the dental structures was measured using Morphometrie10® software, under a 4-fold magnification lens. The ratio between the contact length between the screws and the dental root related to the total length of the screws was calculated. Similarly, measurement of the “bone-to-implant contact” length was assessed, since this is considered to be a quantitative measure for the osseointegration of the device. The “bone-to-implant contact” rate was calculated as follows: screws surface length in contact with bone tissue / (total screws surface length - screws surface length in contact with dental germ or root) (Fig. 3). According to Parfitt et al. 13., measurements of the following parameters were performed using an optical microscope connected with an automatic image analyzer system (BIOCOM, Lyon, France): trabecular bone volume (BV/TV,%), trabecular thickness (Tb.Th, µm), trabecular number (Tb.N), trabecular separation (Tb.Sp, µm). The mineral apposition rate (MAR, µm/day) was determined under UV light on unstained sections (12- µm-thick). These parameters were assessed for the bone area (test zone) located at the apical third of the devices which is the only location where there is sufficient bone for analyses (no germs/root contact) for all the studied devices. A bone area (control zone) located approximately 1.5 cm posterior to the test zone (same location within the bucco-lingual dimensions of the alveolar process) was also analyzed. The test zone and the control zone were both situated 1 to 2 mm buccal from dental germs.

### Statistical analysis

The data were described with the median and the interquartile range. A logistic mixed model was used to estimate the survival rate according to the type of devices used. To test the effect of the type of devices and of their anterior or posterior position on the different outcome results, we used a non-parametric test adapted to repeated measurements and a linear mixed model. The significance level was set as p<0.05. All statistical analysis was performed using R statistical software 2.10.0.

## Results

No complication occurred operatively or post-operatively, no evidence of pain and no infection were detected in any of the animals throughout the study.

### Clinical findings

#### Survival rate

For the 4-week loading period, 27 screws out of 32 remained in the alveolar bone: 14 stainless steel and 13 Ti6Al4V (Table 1). Thus, the survival rate reached 87.5% and 81.3% for stainless steel and titanium, respectively, without statistically significant differences (OR = 0.62, 95% confidence interval 0.08; 4.6., p = 0.64). For the 12-week loading period, 9 out of the 16 screws inserted remained in the alveolar bone; 5 stainless steel and 4 Ti6Al4V. The survival rate was 62.5% for the stainless steel and 50.0% for the titanium devices, without significant differences (OR = 32.8, 95% confidence interval: 0.8; 1326., p = 0.09); however the probability of being retained appears to be higher for stainless steel devices.

**Table 1:** Survival rate and mobility of the miniscrew implants at 4 and 12 weeks after immediate-loading.

Groups	n^a^	Survival rate						Mobility^b^					
		LEONE (stainless steel)			Absoanchor (Ti6Al4V)			LEONE (stainless steel)			Absoanchor (Ti6Al4V)		
		Ant	Post	Total	Ant	Post	Total	Ant	Post	Total	Ant	Post	Total
At 4 weeks													
Group 1	16	4	4	8/8 (100%)	4	2	6/8 (75%)	1	1	2	1	0	1
Group 2	16^c^	3	3	6/8 (75%)	3	4	7/8 (87.5%)	1	1	2	1	0	1
Total	32	7	7	14/16 (87.5%)	7	6	13/16 (81.3%)	2	2	4	2	0	2
At 12 weeks													
Group 2	16^c^	2	3	5/8 (62.5%)	2	2	4/8 (50%)	0	0	0	0	0	0

a number of miniscrew implants inserted ^b^number of mobile miniscrew implants (which survived) ^c^same devices.

#### Survival period

Over both loading periods, 9 devices were lost, 5 in the first 3 weeks and 3 at 7 weeks (Table 2).

**Table 2:** Maximum survival period of the miniscrew implants, and average gingival index and mobility during the loading periods (4 weeks and 12 weeks).

Loading periods	LEONE (stainless steel)				Absoanchor (Ti6Al4V)			
	n*	Survival time*	Average gingival index**	Mobile devices**	n*	Survival time*	Average gingival index**	Mobile devices**
0-4^th^ week								
Group 1	0/8	–	0.69	2	2/8	1 week (n = 1)	0.88	1
						2 weeks (n = 1)		
Group2	2/8	2 weeks (n = 2)	0.62	4^b^	1/8	3 weeks (n = 1)	0.52	1
5–12^th^ week								
Group 2	1/6^a^	7 weeks (n = 1)	0.55	4^c^	3/7^a^	5 weeks (n = 1)	0.46	1^c^
						7 weeks (n = 2)		

* lost devices **during the loading period (for devices which survived to the end of the specified period).

a number of lost devices among the devices which survived after 4 weeks of loading ^b^two of the four mobile devices during the loading period were stable at the end of the 4-week period, ^c^all of the mobile devices were stable at the end of the loading period (12 weeks).

#### Gingival inflammation

The average inflammation index around the retained devices ranged from 0 to 1 (slight inflammation) regardless of the loading period (Table 2). During the first follow-up period (n = 27 survival screws at 4 weeks), the maximal gingival index reached 2 (n = 5 devices) or 3 (n = 4 devices) for a maximum of 7 consecutive days. During the second follow-up period (n = 9 survival screws at 12 weeks), the maximal gingival index reached 2 (n  =  6 devices) for a maximum of 5 days in two animals and for non-consecutive periods spanning over 1 to 3 weeks in a single animal for each of the two devices.

#### Clinical mobility

At 4 weeks, 6 (22.2%) of the 27 retained devices presented abnormal mobility while the 21 (77.8%) remaining devices displayed no mobility (Table 1). This mobility affected both systems and all animals. At 12-weeks, no mobility was present for the 9 devices that had survived; however, 5 of these non-mobile devices (55.6%) presented transient mobility during the loading period (Table 2).

For the 4-week loading period, mobility and gingival inflammation occurred simultaneously in some cases. The periods of severe gingival inflammation were found for mobile devices (4 devices in 3 animals), in cases with visible dental plaque (3 devices in the same animal), and also in cases without mobility or visible dental plaque (2 devices in the same animal). For the 12-week loading period, gingival inflammation was always present with visible dental plaque with (n  =  4 devices) or without mobility (n  =  2 devices). When the periods of moderate or severe gingival inflammation were related to mobility, inflammation appeared either at the same time or after the clinical mobility, with the exception of one case.

### Histological and histomorphometric analysis

For group 1 (4-week loading period), 13 devices from the 16 inserted were analyzed: 8 stainless steel and 5 titanium. The three other devices were either lost or unsuitable for histological examination, following their removal (failed histological sections due to root or germ proximity). For group 2 (12-week loading period), 5 devices were analyzed: 2 stainless steel and 3 titanium. In this last group, among the 16 inserted, 7 were lost and 4 were not considered for histological analysis (failed histological sections).

#### Devices and tooth proximity

Dental germ/root contact equally affected titanium or stainless steel devices. At 4 weeks, 8 devices (61.5%) showed contact (from 9.87 to 37.44%) with a tooth (dental germ/root) or with the soft connective tissue surrounding the tooth (dental sac/periodontal ligament), and from these 8 devices, 2 had direct contact (from 1.6 to 2.3%) with a tooth (dental germ/root). At 12 weeks, 4 devices (80%) showed contact (from 6.31 to 49.45%) with a tooth (dental germ/root) or with the soft connective tissue surrounding the tooth (dental sac/periodontal ligament), and from these 4 devices, 2 had direct contact (from 2.4 to 14.96%) with a tooth (dental germ/root).

#### Bone-screw interface

**Figure 4:**
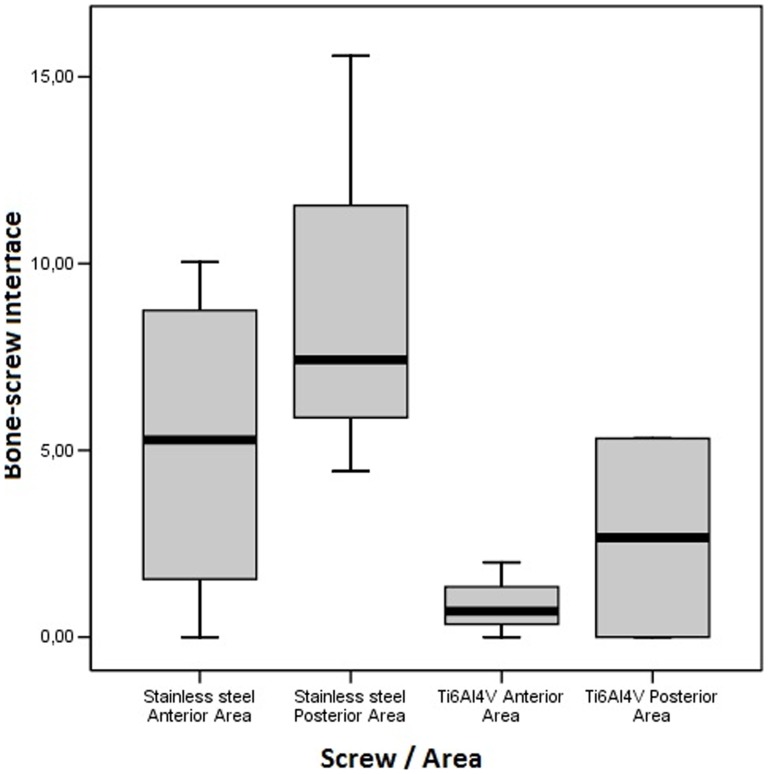
Box plot showing the “bone-screw interface” values after a 4-week loading period of stainless steel and Ti6Al4V screws (median, 25% and 75% percentile). “Bone-screw interface” rate: stainless steel anterior area (5.28% (0.78–9.39)), stainless steel posterior area (7.43% (5.16–13.56)), Ti6Al4V anterior area (0.69%(0.00–2.00)), Ti6Al4V posterior area (2.66%(0.00–5.32)).

**Figure 5:**
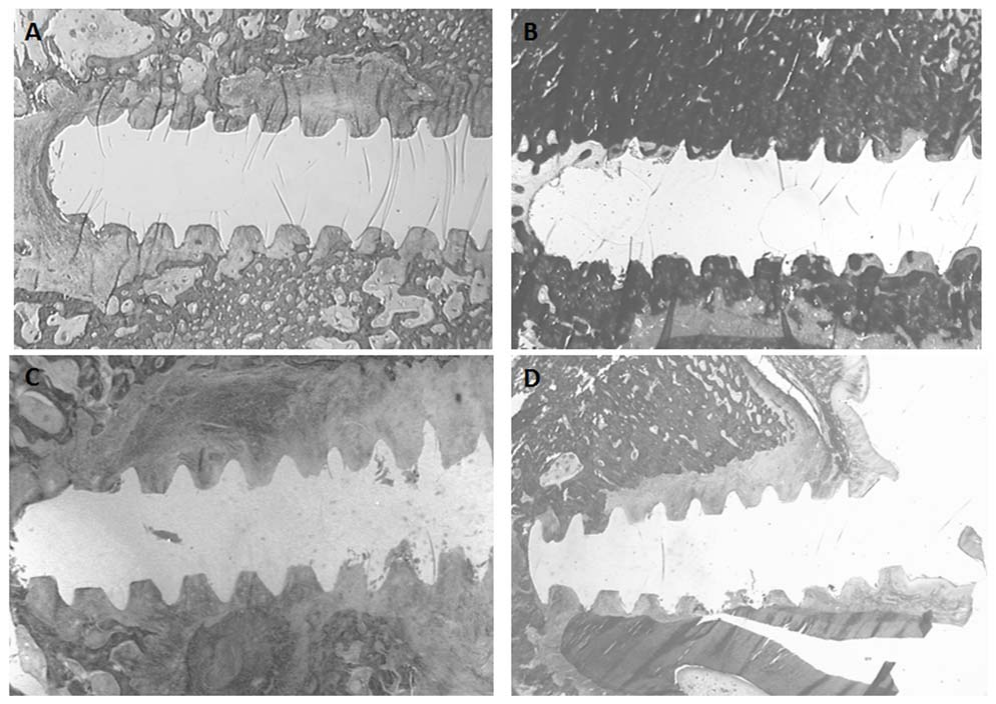
Histological slides (Modified Goldner's Masson Trichrome) of screw devices (x1.6) at 4 weeks; A. (stainless steel device, anterior area), B. (stainless steel device, posterior area), C. (Ti6Al4V device, anterior area), D. (Ti6Al4V device, posterior area).

For the 4-week loading period, regardless of the type of screws or its localization, the median rate (interquartile range) of the “bone-to-implant contact” was between 0.69 (0 – 2.00) and 7.43 (5.16 – 13.56)% (Fig. 4). It ranged from 5.28 (0.78 – 9.39) to 7.43 (5.16 – 13.56)% for the stainless steel devices and from 0.69 (0 – 2.00) to 2.66 (0 – 5.32)% for the titanium devices. According to the localization, the “bone-to-implant contact” rate was from 0.69 (0 – 2.00) to 5.28 (0.78 – 9.39)% in the anterior area and from 2.66 (0 – 5.32) to 7.43 (5.16 – 13.56)% in the posterior area. The stainless steel screws presented greater “bone-to-implant contact” rate than the titanium screws (p = 0.1) as showed in Figure 5. Screws inserted in the posterior area showed the greatest values, without statistically significant differences (p = 0.17). The statistical analysis did not reveal a significant interaction between the type of devices and the localization in the alveolar bone (p = 0.81). For the 12-week loading period, statistical analysis could not be performed since only 5 devices could be considered. Whatever the material or the area, the “bone-to-implant contact” rate varied from 0 to 36.14%. According to the type of devices, this rate varied from 0% to 13.55% (stainless steel devices) and from 0 to 36.14% (titanium devices). Taken all together, over both the 4 and 12-week follow up periods, the “bone-to-implant contact” measurements showed a significant increase for titanium alloy screws as compared to the stainless steel screws.

#### Bone histomorphometric parameters

For the first group, the median values of static and dynamic parameters are indicated in Table 3. Although no statistically significant difference was noted between the two types of devices, some trends were noted between the anterior and posterior zone: while BV/TV, Tb.Th and Tb.N tended to be greater in the posterior zone, the Tb.Sp values appeared to be higher in the anterior area. No statistically significant differences were noted for the mineral apposition rate regarding the kind of screws or the insertion area. Compared with bone in the control area, no significant differences were found (mixed effects logistic regression) for BV/TV (p = 0.23), Tb.N (p = 0.9), Tb.Sp (p = 0.46) and MAR (p = 0.45). Tb.Th values appeared to be greater for bone in the vicinity of the screws than for bone in the control area, especially in the anterior zone (p = 0.05).

**Table 3:** Static and dynamic parameters of bone tissue nearby the devices, after a 4-week loading period (median and interquartile range).

Bone parameters	Devices and localisation				Statistical analysis^a^	
	LEONE (Stainless steel)		Absoanchor (Ti6Al4V)		p-values	
	Anterior	Posterior	Anterior	Posterior	Devices	Area
BV/TV (%)	24.18	35.79	19.93	44.46	0.89	0.12
	(7.64–35.77)	(27.52–44.88)	(18.79–24.10)	(31.06–57.86)		
Tb.Th ( µm)	104.98	123.07	107.07	119.64	0.92	0.26
	(76.36–114.95)	(94.01–125.05)	(75.38–107.28)	(102.71–136.57)		
Tb.N	2.74	3.01	2.25	3.63	1.00	0.21
	(0.98–3.11)	(2.23–4.69)	(1.75–2.64)	(3.02–4.24)		
Tb.Sp ( µm)	276.55	224.75	337.84	163.74	0.91	0.12
	(208.92–1929.48)	(122.64–326.45)	(302.80–462.87)	(99.47–228.00)		
MAR ( µm/day)	1.90	2.20	1.66	1.82	0.60	0.50
	(1.58–2.46)	(1.62–3.17)	(1.62–1.84)	(1.62–2.02)		

a Mann-Withney non parametric test.

For the second group (12-week loading period) and despite the small sample size, median values for BV/TV were 5.76% in the vicinity of the stainless steel screws and 20.54% around the titanium screws. Values for Tb.Th were 52.47 and 82.18 µm; for Tb.N the values were 1.09 and 2.4; for Tb.Sp they were 895.08 and 377.3 µm (for the bone areas around the stainless steel and titanium devices respectively). The median values for the mineral apposition rate reached 2.4 µm/day and 1.87 µm/day around the stainless steel and titanium devices respectively.

## Discussion

The use of screws for orthodontic anchorage requires that the devices remain stable during the treatment time in order to withstand the orthodontic forces 14. Stability is therefore required immediately following loading. Most miniscrew implants are made of titanium or titanium alloys, but some manufacturers have proposed stainless steel miniscrew implants. Stainless steel devices present better mechanical properties as they reach the load at the failure at values that are twice as high as titanium 15. However, they are known to induce less bone interface than titanium or titanium alloy devices. The behavior of stainless steel screws placed in bone tissue is well-known, as it is the most common material used in orthopedics. However, according to our knowledge, no previous studies have assessed the survival rate, the stability and the bone tissue response around stainless steel screws for orthodontic anchorage.

To better control the screws placement within the alveolar bone, a flap was systematically raised for both systems of devices.

### Animal model

In this study, we used the porcine model, an animal model which is close to humans in clinical practice in terms of bone anatomy, healing process and bone tissue remodeling 16., 17., as well as for dental occlusion and breeding characteristics 18–20. Furthermore, histological similarities have been reported for periodontal diseases in pigs and humans 21. Dogs are another animal model commonly used for the study of bone response to dental implants or screws, but as they are domestic animals, ethical considerations prohibited their use in this study that required growing animals. Indeed, although most orthodontic patients are young, few studies have assessed the survival rate or bone tissue response around screws for orthodontic anchorage in adolescents 22–25. or growing animals 26. However, bone microarchitecture 27., bone density 28. and remodeling differ significantly between growing and adult patients 29. Three-month-old pigs present a complete deciduous dentition 30. and allow for studying growing alveolar bone reaction after 12 weeks without eruption of permanent teeth near the assessed devices.

### Histological examination

Screws are usually placed between dental roots in clinical orthodontic practice. Alveolar bone located between teeth and devices may have a specific reaction to orthodontic forces due to its small volume. Therefore, we decided, on one hand, to place the devices near the dental structures and, on the other hand, to characterize this bone volume. In the literature, devices inserted close to teeth are systematically excluded 31., especially because of difficulties involved in preparing such samples in histology. This explains why some samples were unsuitable for histological examination in the present study. Nevertheless, the number of samples was sufficient to obtain statistically significant data for the 4-week group and interesting data for the 12-week group. Further studies are needed to characterize this specific alveolar bone located between loaded orthodontic screws and teeth and to complete the data of the present study.

### Clinical findings

The survival rate of the devices was high at 4 weeks (84.4%) and decreased markedly at 12 weeks (56.25%). The latter result is coherent for a growing model, as young age is considered to be a risk factor for the clinical retention of screws in orthodontic patients 23–25,32,33. Lower bone quality and maturation in young patients 25. can prevent effective primary stability of the device 34,35. To increase the presence probability of such devices in growing bone, it may be advisable for the clinician to increase the healing period before loading in young patients and to avoid immediate loading. A greater survival rate (63.8% after 6 months) for orthodontic screws has been reported in adolescents 25. but the protocol involved a 1-month healing period before loading. Differing opinions have been reported regarding the effect of the healing period before loading; immediate loading has been considered as either a risk factor for the survival rate of the devices 36. or a means to increase mechanical stability 37. In the growing dog model, Vande Vannet et al. 26. reported a 60% survival rate at 6 weeks for Ti6Al4V devices; the retention rate decreased by 45% at 12 weeks and the authors used a 200 g-load in their study. Thus our results fall within the range of previously published results in growing models. Another finding in our study shows a differential susceptibility to the loss of the devices in some animals that exhibit a higher devices loss rate. At 12 weeks, one animal had lost all devices and another had lost 75% of them. Such observations have been reported using large animal 31. In our study, statistical tests were chosen so as to take into account the host effect, i.e. several screws were inserted in each animal.

Mobility of the devices displayed some variation over the follow-up period and it is noteworthy that some of the devices retained at 12 weeks exhibited transient mobility at 4 weeks. It is likely that bone remodeling occurred between 4 and 12 weeks, leading to a final stabilization. Indeed, 55.6% of the devices were lost during the first three weeks (critical period for orthodontic screws success 38.), and 33.3% were lost during the 8^th^ week.

### Histomorphometric analysis

In our study the “bone-to-implant contact” percentage was low after 4 weeks (median rate < to 7.43%), and increased between 4 and 12 weeks, with a maximum value of 36.14% (Ti6Al4V devices). This is close to the results of a previous study on titanium screws 39. which revealed a “bone-to-implant contact” rate equal to 36.3% after 12 weeks of immediate loading in growing dogs. The increase of “bone-to-implant contact” rate over a period of time, as shown in our study, confirmed results of previous studies 3,31,40,41. Some authors have reported a minimal integration index for the stability of orthodontically-loaded implants of 5% 39,42. or 10% 43. In the present study, the 5% threshold was obtained after 4 weeks with the stainless steel devices, thus suggesting a better survival rate. This may be related to enhanced primary stabilisation with the stainless steel as opposed to the titanium devices, due to their morphological characteristics. Different shapes for the devices may account for this observation; the torque values of cylindrical-shaped screws differ from those of cylindro-conical devices 44,45. Further investigations are necessary to understand the role of device morphology in relation to bone response and to confirm the better clinical results of the stainless steel screws.

In our study, no differences in bone response were found between both devices, similar to previous reports 46. which studied bone healing around buried and unloaded stainless steel and titanium orthopaedic screws inserted into the calvaria of dogs. Temporary modifications (increased trabecular thickness) of the bone surrounding the devices were noted at 4 weeks in the anterior region and were not found again at 12 weeks. The porcine mandibular bone does not remain stable during the growing process and shows a specific density distribution in the jaw according to the age of the animals 47.; the response to strain also varies according to the bone architecture 48. The body of the porcine mandible has predominantly sagittal growth 30., with no evidence of interstitial growth in the bone and only appositional growth in the condyle area and the posterior border of the mandible 49. However, this data does not provide information about the anterior versus posterior alveolar bone areas. It has been suggested that bone density increases when the devices are loaded compared to when they are not loaded 35,50., or that immediate loading increases the bone density around dental implants 51. Animal feeding can also interact with bone density 52. and a hard diet would favor increased trabeculations 53. In the growing animal model, increased bone density is most probably an adaptation mechanism to the stress induced by loading.

## Conclusion

No significant differences in bone response were shown between stainless steel and titanium alloy devices in our study. Nevertheless, the 5% threshold of “bone-to-implant contact” was obtained after 4 weeks with the stainless steel devices, leading to a better survival rate. The survival rate of immediately loaded screws is quite low in a growing model and the first three weeks after loading may be a critical period for device retention. In our growing model, and for bone with low density, it is likely that increased trabecular thickness is the bone response to the stress induced by loading.
